# Periodic static compression of micro-strain pattern regulates endochondral bone formation

**DOI:** 10.3389/fbioe.2024.1356135

**Published:** 2024-03-27

**Authors:** Pengzhen Cheng, Xueyi Zhao, Meige Han, Yaping Zhuang, Fenru Ning, Yaqian Hu, Weiguang Lu, Sheng Miao, Chengxiang Zhao, Liyuan Jia, Xue Hao, Meng Sun, Junxiang Wang, Fulin Chen, Liu Yang, Qiang Jie

**Affiliations:** ^1^ College of Life Sciences, Northwest University, Xi’an, China; ^2^ Pediatric Orthopaedic Hospital, Honghui Hospital, Xi’an Jiaotong University, Xi’an, China; ^3^ Department of Orthopedics, Xijing Hospital, Fourth Military Medical University, Xi’an, China; ^4^ Xi’an Key Laboratory of Skeletal Developmental Deformity and Injury Repair, Xi’an, China; ^5^ Department of Orthopaedics, Shanghai Key Laboratory for Prevention and Treatment of Bone and Joint Diseases, Shanghai Institute of Traumatology and Orthopaedics, Ruijin Hospital, Shanghai Jiao Tong University School of Medicine, Shanghai, China; ^6^ Department of Neonatology, The First Affiliated Hospital of Xi’an Jiaotong University, Xi’an, China; ^7^ Research Center for Skeletal Developmental Deformity and Injury Repair, School of Life Science and Medicine, Northwest University, Xi’an, China

**Keywords:** periodic static compression, micro-strain, critical bone defects, endochondral bone formation, biomaterials

## Abstract

**Introduction:** Developmental engineering based on endochondral ossification has been proposed as a potential strategy for repairing of critical bone defects. Bone development is driven by growth plate-mediated endochondral ossification. Under physiological conditions, growth plate chondrocytes undergo compressive forces characterized by micro-mechanics, but the regulatory effect of micro-mechanical loading on endochondral bone formation has not been investigated.

**Methods:** In this study, a periodic static compression (PSC) model characterized by micro-strain (with 0.5% strain) was designed to clarify the effects of biochemical/mechanical cues on endochondral bone formation. Hydrogel scaffolds loaded with bone marrow mesenchymal stem cells (BMSCs) were incubated in proliferation medium or chondrogenic medium, and PSC was performed continuously for 14 or 28 days. Subsequently, the scaffold pretreated for 28 days was implanted into rat femoral muscle pouches and femoral condylar defect sites. The chondrogenesis and bone defect repair were evaluated 4 or 10 weeks post-operation.

**Results:** The results showed that PSC stimulation for 14 days significantly increased the number of COL II positive cells in proliferation medium. However, the chondrogenic efficiency of BMSCs was significantly improved in chondrogenic medium, with or without PSC application. The induced chondrocytes (ichondrocytes) spontaneously underwent hypertrophy and maturation, but long-term mechanical stimulation (loading for 28 days) significantly inhibited hypertrophy and mineralization in ichondrocytes. In the heterotopic ossification model, no chondrocytes were found and no significant difference in terms of mineral deposition in each group; However, 4 weeks after implantation into the femoral defect site, all scaffolds that were subjected to biochemical/mechanical cues, either solely or synergistically, showed typical chondrocytes and endochondral bone formation. In addition, simultaneous biochemical induction/mechanical loading significantly accelerated the bone regeneration.

**Discussion:** Our findings suggest that microstrain mechanics, biochemical cues, and *in vivo* microenvironment synergistically regulate the differentiation fate of BMSCs. Meanwhile, this study shows the potential of micro-strain mechanics in the treatment of critical bone defects.

## 1 Introduction

The repair of critical bone defects caused by high-energy trauma and disease is a common clinical challenge. Previous studies have reported that the approach based on endochondral osteogenesis is more effective in treating critical bone defects compared to intramembrane osteogenesis strategies (Thompson et al., 2016; Bernhard et al., 2017; Xie et al., 2022). This technology based on endochondral ossification has been proposed as a strategy to mimic development, with some similarities to the process of bone development. However, replicating the developmental microenvironment, which includes constructing bioactive materials, supplementing biochemical factors, and improving the mechanical loading patterns, remains problematic.

Under physiological conditions, growth plate cartilage is subjected to compressive forces from the articular surface and tensile forces from the tendons and ligaments, resulting in deformation and micro-movement of the extracellular matrix (ECM) surrounding the chondrocytes (Zhang et al., 2022). A previous study showed that mechanical loading applied to the epiphysis produced longer skeletal elements and increased bone density (Foster, 2019). Despite many studies reporting on the regulation of bone and cartilage regeneration by mechanical loading (Uzieliene et al., 2021), there is no consensus on the paradigm and characteristics of mechanical loading for bone/cartilage engineering. Indeed, the outcome of mechanical intervention largely depends on the loading parameters (Uzieliene et al., 2021). As mentioned in the literature, the abnormal high stress is mainly concentrated on the articular surface and the secondary ossification center (SOC), thus leading to the deformation of the articular cartilage. However, due to the shock-absorbing effect of SOC, the endochondral ossification site is maintained in the micro-mechanical stimulation environment (Xie et al., 2020). However, the regulatory effect of micro-mechanical loading on endochondral bone formation has not been investigated.

Sloas DC et al. recently designed a mechanical force receptor with high sensitivity. Cells expressing these receptors could distinguish extracellular tension at the pico Newton level (Sloas et al., 2023), which demonstrate the potential of cells as micro-mechanical sensors. BMSCs are considered to be classical regenerative cells for bone and cartilage repair (Uzieliene et al., 2021), while BMSCs are mechanosensitive and have the potential to respond to the mechanical signals of the microenvironment (Wang et al., 2022b; Sun et al., 2022).

In this study, we designed a simple *in vitro* mechanical loading model and performed periodic static compression (with 0.5% strain) on the hydrogel scaffold loaded with BMSCs. Subsequently, the chondrogenic differentiation and hypertrophic maturation status of BMSCs were observed. To further assess the “biochemical-mechanical memory” and bone-repair capacity in ectopic and *in situ* niches, the pretreated scaffolds were transplanted into rat femoral muscle pouches and femoral condylar defect sites. The results demonstrate the multivariate regulatory role of biochemical/mechanical cues and local niche on the differentiation lineage of BMSCs, providing a potential regenerative strategy for critical bone defects.

## 2 Materials and methods

### 2.1 Preparation of the HAMA-Alg hydrogel

Add 10 mL of PBS buffer to fully dissolve lithium phenyl (2,4,6-trimethylbenzoyl) phosphinate (LAP, photoinitiator) in dark, add hyaluronic acid methacryloyl (HAMA, 2% w/v) to LAP solution, heat and stir at 40°C for 1-2 h until HAMA fully dissolved and then sterilized. Add the sterile sodium alginate (Alg, 2% w/v) powder to the HAMA solution with full stirring until dissolved, a mixture of 2% HAMA-2% Alg was obtained after bubble removal by centrifugation.

The purification and incubation steps of rat BMSCs refer to our previous study (Cheng et al., 2018). Briefly, BMSCs were isolated from the femoral bone marrow of 2-week-old wild-type rats. The medium (α-MEM+10% FBS+1% penicillin-streptomycin mixture) was changed every 48 h to remove the dead and nonadherent cells.

Rat BMSCs were trypsinised then resuspened, and fully mixed at 1 × 10^7^ cells/mL with the hydrogel mixture as described above, and it was further injected into the cylindrical mold and solidified by blue light for 10 s. After demoulding, the hydrogel cylinder was immersed in 2% CaCl_2_ solution for secondary crosslinking for 5–10 min, and the HAMA-Alg composite hydrogel loaded with BMSCs was obtained after full washing.

In this study, molds of different sizes were prepared using polycaprolactone (PCL), with a diameter of 10 mm for *in vitro* experiments and 3.5 mm for *in vivo* experiments. The height of all molds was 5 mm.

### 2.2 Mechanical test

The samples were made into a cylinder with a diameter of 10 mm and a height of 5 mm. A preload of 0.5 N was applied to the hydrogel to fix the sample (prevent sample sliding), run the test procedure at the speed of 2 mm/min and terminate when 35% strain. The mechanical testing machine (TA, ELF3220, United States) automatically recorded the real-time data of “Load and Disp,” further calculated the stress, strain and elastic modulus data manually and plotted the stress-strain curve.

### 2.3 Live-dead staining

The Calcein-AM/Propidium Iodide (PI) dual staining kit (DOjinDO, C542, Japan) was used to label and identify live and dead cells. Calcein-AM removes AM in the presence of esterase within living cells, producing calcein and emitting green fluorescence. PI can be embedded into the DNA double helix of dead cells, emitting red fluorescence. Therefore, green fluorescence represents live cells, and red fluorescence represents dead cells. The operation details were performed according to the instructions.

### 2.4 Microstructure and composition

The structural and elemental analysis of lyophilized samples were performed using SEM and energy dispersive x-ray spectroscopy (EDX, Hitachi-S3400N, Japan). EDX captures information through Si (Li) detector. The test condition was 15 KeV, the working distance was 10 mm, the magnification was ×200, the process time was 60 s, and the spectrum range was 0–20 KeV.

### 2.5 Periodic static compression

Periodic static compression experiments were performed in proliferation medium and chondrogenic medium. The proliferation medium contained 15% fetal bovine serum (Gibco, United States), and the chondrogenic medium contains several active ingredients, such as ITS (Insulin-Transferrin-Selenium), TGF-β3 and dexamethasone (Cyagen, RAXMX-90041, China). HAMA + Alg scaffolds were randomly divided into four groups: control group (CON, proliferation medium), periodic static compression group (PSC, proliferation medium), chondrogenic differentiation group (CM, chondrogenic medium), chondrogenic differentiation and periodic static compression group (CM + PSC, chondrogenic medium). The HAMA + Alg hydrogel cylinders were incubated in a 24-well plate, and the sterilized titanium block adapted to the 24-well plate was covered on the hydrogel surface. The titanium block was a custom-made medical metal (NATON, China), with a composition of Ti-6Al-4V, and the reference standard was “GB/T 3620.1-2007.” The titanium blocks were non-toxic and did not affect cell survival and metabolism. In addition, titanium blocks were disinfected daily with high-pressure sterilizers and strict aseptic procedures were followed. According to the measurement and calculation, the pressure on the hydrogel scaffold was about 2.3 kPa, and the resulting strain was about 0.5%. The samples were loaded for 20 min and then unloaded for 20 min. This loading-unloading process was a cycle, eight consecutive cycles were performed per day, leaving the remaining time for conventional incubation without loading. Samples were harvested on days 7, 14, and 28 for subsequent testing.

### 2.6 Histological analysis

The samples were fixed in 4% paraformaldehyde (BOSTER, China) for 72 h. After dehydration for 24 h, the samples were embedded in optimal cutting temperature compound (OCT, sakura, United States), and then performed frozen sectioning (LEICA, Germany) with a slice thickness of 8 μm. Random sections from each group were stained with HE, Safranin-O and Fast Green (Solarbio, G1371, China). Detailed operation was conducted according to the product instructions. Briefly, the frozen sections were returned to room temperature, and rinsed with tap water and then added with working solution for staining. Finally, the sections were dehydrated in alcohol and sealed with resinene. After histological staining, images were taken under a ×20 or ×40 objective with a resolution of 4,080 × 3,072. The image was taken by a light microscope (Olympus, BX53, Japan).

### 2.7 Cytoskeleton staining

We used FITC labeled Phalloidin (abclonal, RM02836, China) to visualize the cytoskeleton. Phalloidin specifically binds to filamentous actin (F-actin) in eukaryotic cells. After incubating FITC-Phalloidin with cells for 30–60 min, the morphology and distribution of F-actin were observed by green fluorescence. The operation details were performed according to the instructions. Briefly, random sections were fixed with 4% paraformaldehyde for 15 min and subsequently washed three times with PBS containing 0.1% TritonX-100. The staining solution was added and incubated for 30 min, then repeated the above washing steps. The images were captured using a fluorescence microscope (Olympus, BX53, Japan).

### 2.8 Immunofluorescence and immunohistochemical staining

The procedures were describled in our previous study (Cheng et al., 2022). The primary antibody information was as follows: anti-SOX9 (Abcam, ab185966, UK, 1:200), anti-Collagen Ⅱ (COL Ⅱ, Santa, sc52658, United States, 1:100), anti-Collagen X (COL X, Abclonal, A18604, China, 1:100), anti-MMP13 (Abcam, ab39012, UK, 1:200), anti-SP7/Osterix (Abcam, ab209484, UK, 1:200).

We extracted the positive regions of immunofluorescence staining (COL X and MMP13) using Image J software (Version 1.53 h) and further calculated the average positive expression area of each group. Finally, the average positive area of all biological replicate samples was collected for statistical analysis. It is worth noting that since antibodies detect secreted proteins rather than transcription factors, the area of the nucleus is deducted.

In addition, Ki67 is involved in the regulation of chromosome segregation and regulation of mitotic nuclear division. Therefore, it is used to label cells in the proliferative cycle. In this study, the Ki67 antibody (Abclonal, A20018, China, 1:100) was used to visualize the proliferating cells. The number of Ki67-positive cells was counted using the Image J software (Version 1.53 h).

### 2.9 Biological nanoindentation

The hydrogels of each group pretreated for 28 days *in vitro* were washed clean with PBS. The detection procedure was 10 × 10 grid (100 indents), the stiffness of the probe was 0.5 N/m. In addition, the distance between adjacent indentations was 2 μm and the loading speed was 10 μm/s. Hardness detection was performed using a bio-nanometer indentation instrument (Optisc11, Piuma, Netherlands).

### 2.10 Animals

Sprague-Dawley (SD) rats (male, 14 weeks old, 250 ± 20 g) were obtained from the experimental animal center of the Fourth Military Medical University. All experimental animals were maintained in the animal facility of the experimental animal center of the Xijing Hospital. Animal surgical procedures are approved by the Ethics Committee for Animal Research of Fourth Military Medical University (IACUC-20210242).

### 2.11 Muscle pouch transplantation model

The animal surgical procedures were consistent with those described in our previous study (Miao et al., 2022). The brief description is as follows. Rats were anesthetized by intraperitoneal injection of pentobarbital sodium (30 mg/kg, Merck, Germany). A hydrogel scaffold was implanted into the posterior gluteal muscle pouch (approximately 12 mm) of 3 rats in each group. The muscle pouch could completely encapsulate the scaffold to ensure that the scaffold is not squeezed out during muscle contraction. The experimental rats were euthanized at 10 weeks after implantation to harvest the implants.

### 2.12 Rat femoral condyle defect

The Wild-type SD rats were randomly divided into four groups with 20 rats in each group. The experimental rats were anesthetized by intraperitoneal injection of 2% w/v pentobarbital sodium. A standard cylindrical bone defect (3.5 mm in diameter and 5 mm in depth) was made on the medial side of the femoral condyle using a bone drill. During the surgery, physiological saline was used to cool the drill bit, flush bone fragments and blood.

The samples from the four groups (CON, PSC, CM, CM + PSC) were pretreated *in vitro* for 28 days (described in section 2.5) and then cut into cylindrical grafts with 3.5 mm diameter and 5 mm height using a hollow mold. Subsequently, we implanted them into the bone defect site before suturing the muscles and skin. Antibiotics (50 kU/kg penicillin) were injected intramuscularly for 3 consecutive days after surgery to prevent infection. The chondrogenesis and bone defect repair were evaluated 4 or 10 weeks post-operation.

### 2.13 Sirius red staining

The sections of each group were stained with Sirius red according to the instructions of the staining kit (Solarbio, G1470, China), and the distribution of type I and type III collagen at the defect site was evaluated by polarizing microscope imaging system (OLYMPUS, BX53, Japan). Type I collagen was colored orange or red and type III collagen was green under the polariscope.

### 2.14 Calcein-alizarin red double fluorescent labeling

The operational details were described as previous study (Liu et al., 2020).The reagents information were calcein (Sigma-aldrich, C0875, United States, 25 mg/kg) and alizarin red (Sigma-aldrich, A5533, United States, 30 mg/kg). Both fluoresceins were deposited at the osteogenic site at the injection time point, and the distance between the two fluorescent lines was the amount of bone formation at the injection interval. Subsequently, the daily amplification distance was calculated, which is the mineral apposition rate (MAR, μm/day). It is worth noting that we measured the fluorescent marker at the junction of the undegraded hydrogel and new bone, that is, the front edge of new bone formation.

### 2.15 Micro-CT analysis

Micro-CT imaging was performed as described previously (Cheng et al., 2022). The samples from each group were scanned by high-resolution micro- CT system (BRUKER, SkyScan1276, Germany). The source voltage was 65 kv, the source current was 200 μA, and the resolution was 8 μm. The 3D reconstruction and data analysis were performed via NRecon (v2.0.01) and CTAN (1.20.8–64bit) software. We analyzed the indicators of new bone formation at the defect site, including the percentage of bone volume (BV/TV), the trabecular number (Tb.N), the trabecular thickness (Tb.Th), and the trabecular separation (Tb.Sp).

### 2.16 Histological score of bone defect repair

The sections (from 4 weeks post operation, *n* = 6) were stained with safranin O-fast green and HE. The regeneration status of bone defects was evaluated by referring to the histological scoring system previously reported by (Fortier et al., 2002; Madry et al., 2020). Briefly, 0 represents a close to normal histological appearance and 3 represents a severe defect or completely unrepaired histological appearance.

Furthermore, the histological scoring data were used to further analyze the correlation between the morphology and number of chondrocytes and interface integration status, as well as hydrogel degradation.

### 2.17 Statistical analysis

All data were presented as the mean ± SD. One-way ANOVA followed by multiple comparisons test was used to compare differences when multiple groups were compared with each other. The Pearson correlation coefficient was used to evaluate the correlation between the variables. The GraphPad Prism 9.3 package (GraphPad Software, CA, United States) was employed for analysis. Statistical significance was preset at *p* = 0.05.

## 3 Results

### 3.1 Preparation and characterization of BMSC-loaded hydrogels

**FIGURE 1:**
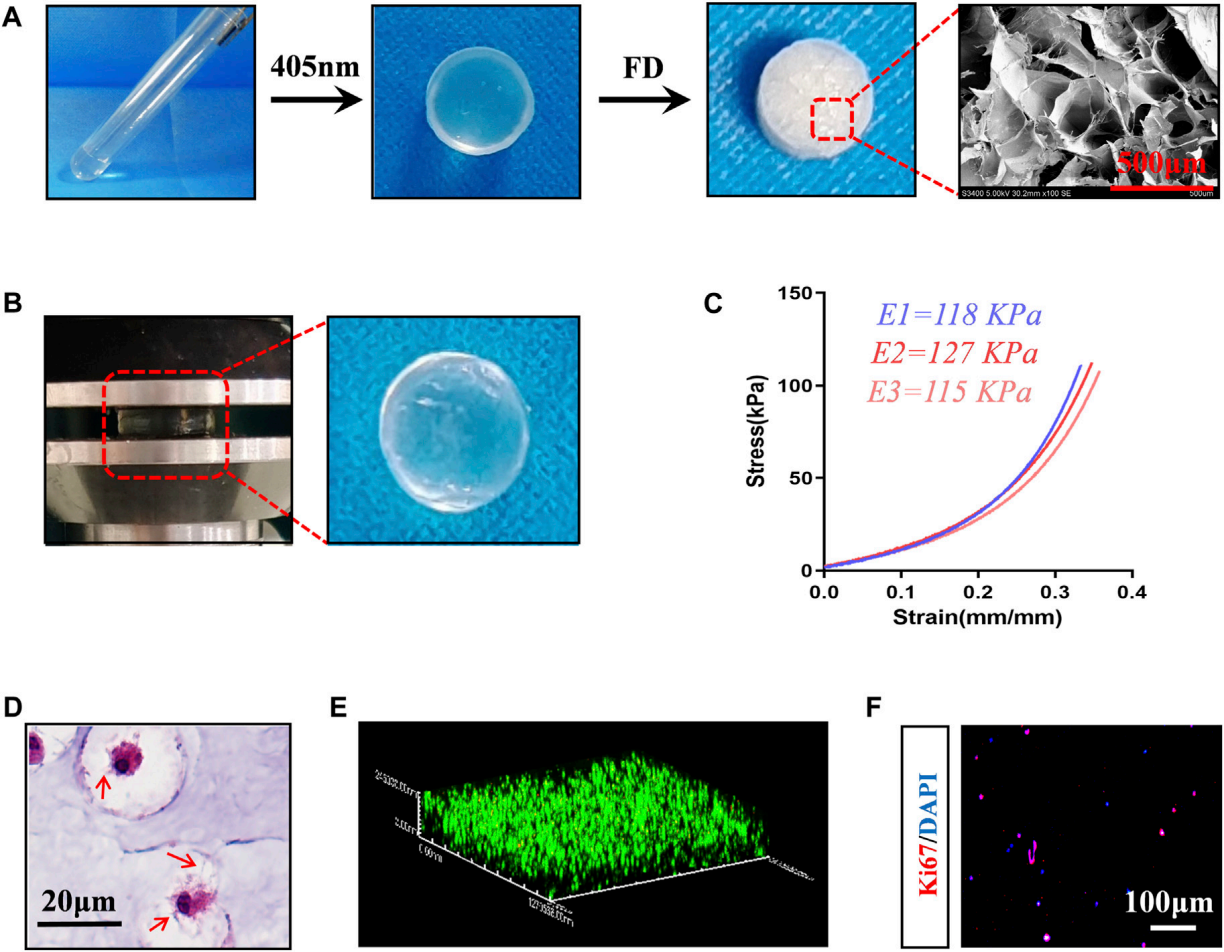
Preparation and characterization of composite scaffolds loaded with BMSCs. **(A)** Preparation procedure of hydrogel scaffold, Photocrosslinking of hydrogels -- Freeze drying (FD) -- Microstructure image. **(B)** Mechanical testing (35% strain) and the appearance of the loaded hydrogels. **(C)** Stress-strain curve, E1-E3 represents repeated data of elastic modulus. **(D)** HE staining images of BMSCs-loaded scaffolds incubated *in vitro* for 7 days. The red arrow represents the pseudopodia of BMSCs. **(E)** 3D reconstruction image of live-dead staining, red represents dead cells, green fluorescence represents living cells. **(F)** Ki67 staining image.

To construct cell-carrying scaffolds with good mechanical properties and biocompatibility, we prepared HAMA-Alg dual-network hydrogels. The results showed that the scaffold has plasticity and a relatively uniform microstructure, with a pore size of approximately 200–500 μm (Figure 1A). The scaffold remained undamaged even after performing the axial compression load that caused 30% strain (Figure 1B). The stress-strain curves of the three repeated samples showed a smooth appearance without an obvious inflection point, indicating the strong capacity of the scaffold to store energy and maintain space. The elastic modulus of the samples was greater than 110 kPa, suggesting that it can meet the mechanical requirements as a filling material (Figure 1C).

When rat BMSCs (Supplementary Figure S1) were loaded into HAMA-Alg and incubated *in vitro* for 7 days, it was found that the pseudopodium protruded and formed a special “stem cell niche,” which may be attributed to paracrine and hydrogel degradation (Figure 1D). Meanwhile, most BMSCs survived in the scaffold, and some cells had the potential for proliferation (Figures 1E, F).

### 3.2 Proliferation and morphology of the BMSCs are affected by PSC based on the micro-strain pattern

A straightforward *in vitro* loading test model was created, which continually applied static compression load to the scaffold in a 24-well plate using a titanium alloy block (Supplementary Figure S1C).

**FIGURE 2:**
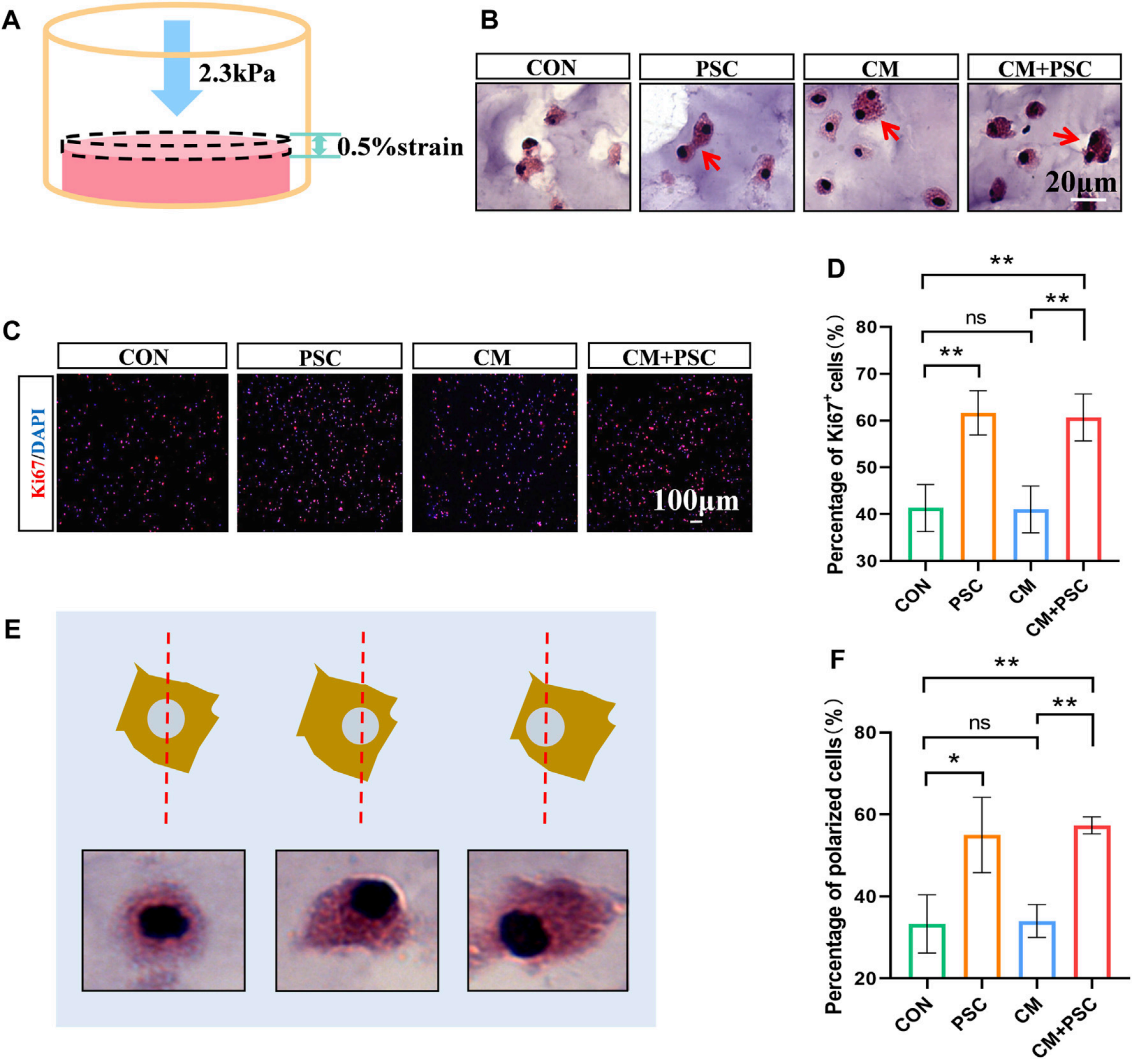
Effects of periodic static compression on proliferation and polarity of BMSCs. **(A)** Mechanical loading diagram. The surface pressure of the hydrogel is 2.3 kPa, and the strain is 0.5%. **(B)** HE staining images of hydrogel after biochemical/mechanical intervention for 7 days (CON, control; PSC, periodic static compression; CM, chondrogenic medium; CM + PSC, chondrogenic medium and periodic static compression). The red arrow represents dividing and proliferating cells. **(C)** Ki67 staining image. **(D)** Statistical data of the proportion of Ki67 positive cells. **(E)** Schematic diagram of polar cells. Nuclear center location was used as a reference. **(F)** Statistical results of percentage of polar cells. Data were represented as the mean ± SD. *p < 0.05, **p < 0.01.

Calculations revealed that the stress applied on the scaffold was 2.3 kPa, and the resulting strain was approximately 0.5% (Figure 2A). The BMSCs in the scaffold were well-developed after incubation *in vitro* for 7 days, with a few cells dividing (Figure 2B). According to the results of Ki67 staining, PSC significantly increased BMSC proliferation, regardless of whether proliferation or chondrogenic induction media was used (Figures 2C, D). Since mechanical force-induced polar distribution of the cytoskeleton is related to cell migration and adhesion, to understand whether cells respond to external forces, the number of “polarized cells” with unbalanced cytoplasm distribution were counted, using the nucleus as a reference center. The findings demonstrated that PSC significantly raised the number of polarized cells without the need for biochemical cues such as chondrogenic stimulation (Figures 2E, F).

### 3.3 Effects of PSC on chondrogenic differentiation of BMSCs

**FIGURE 3:**
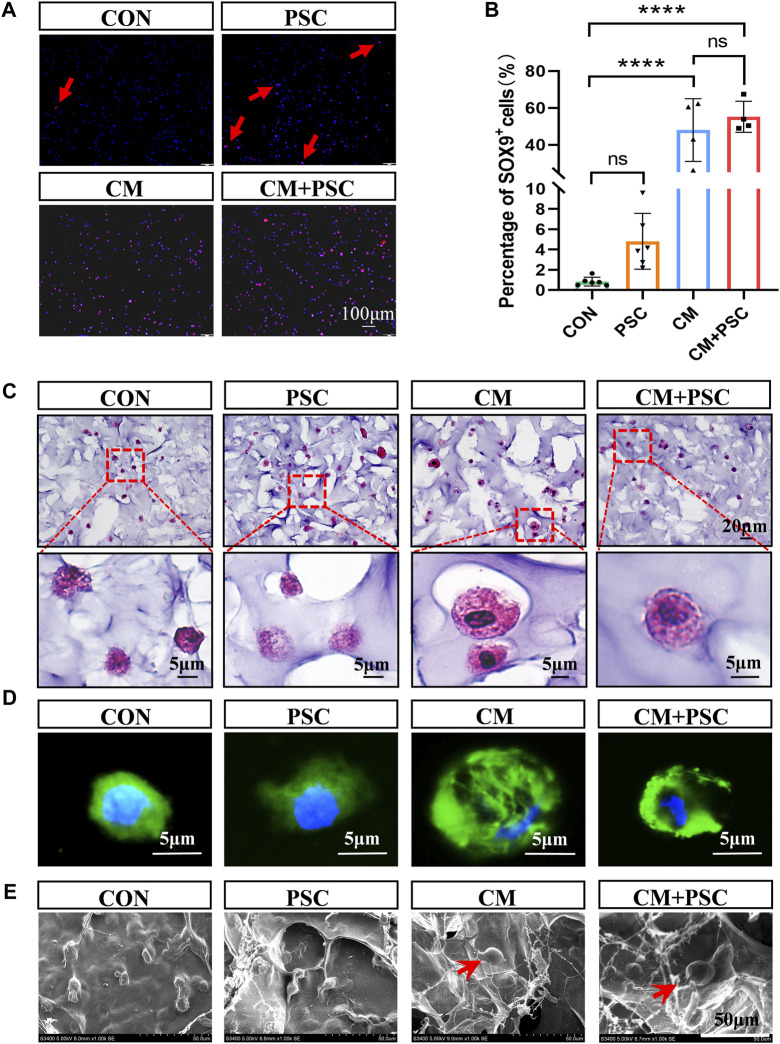
Effects of biochemical/mechanical cues on chondrogenic differentiation and morphology of BMSCs. **(A)** SOX9 immunofluorescence staining image of the composite scaffold stimulated for 14 days *in vitro*. Red arrow represents SOX9 positive cells. **(B)** Statistical results of percentage of SOX9^+^ cells. **(C)** HE staining image of the composite scaffold stimulated for 28 days *in vitro*. **(D)** Phalloidin staining image. **(E)** Cell morphology captured by SEM. The red arrow points to hypertrophic chondrocyte-like cells. Data were represented as the mean ± SD. ****p < 0.0001.

In the PSC group, approximately 4% of the SOX9-positive cells were visible after 14 days of loading in the proliferative medium, and the proportion of COL Ⅱ^+^ cells increased noticeably (accounting for 52% of the total cells). However, regardless of whether PSC was performed, the number of cells expressing SOX9 and COL Ⅱ increased dramatically under chondrogenesis induction (Figures 3A, B; Supplementary Figures S2A, B). In summary, inducing chondrogenic differentiation of BMSCs by biochemical factors such as TGF-β3 and ITS is more effective. In addition, the application of PSC alone stimulated the chondrogenic differentiation of BMSCs but with low induction efficiency; the chondrogenic potential of the BMSCs was significantly increased by the synergistic application of biochemical factors and mechanical loading in the short term.

Surprisingly, some sizable cells that resembled hypertrophic chondrocytes were observed in the CM and CM + PSC groups after 28 days of *in vitro* treatment. The morphology, cytoskeleton, and electron microscopy images of these cells were considerably different from those of cells in the proliferative medium (Figures 3C–E). These cells were significantly expanded in volume, larger than ordinary cells and had a spherical appearance. In addition, the image of the cytoskeleton showed that their nucleocytoplasmic ratio is relatively small. Whether biochemical induction and mechanical loading promote the hypertrophy and maturation of ichondrocyte requires further evidence.

### 3.4 Effects of biochemical/mechanical cues on hypertrophy and mineralization of ichondrocytes

**FIGURE 4:**
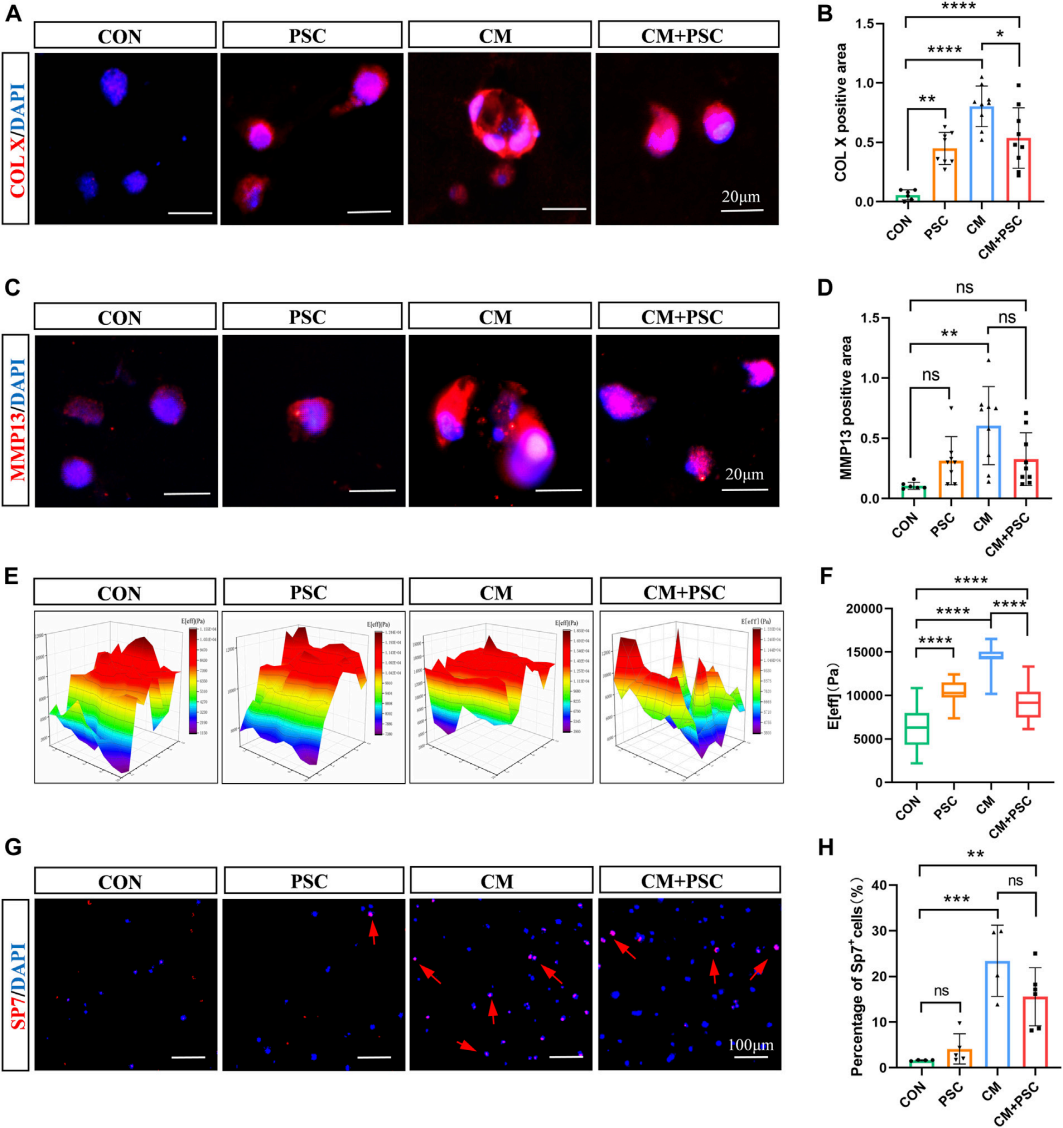
Effects of biochemical/mechanical cues on hypertrophy and mineralization of ichondrocytes. **(A)** COL X immunofluorescence staining image of the composite scaffold stimulated for 28 days *in vitro*. **(B)** Statistical results of COL X positive area. **(C)** MMP13 immunofluorescence staining image of the composite scaffold stimulated for 28 days *in vitro*. **(D)** Statistical results of MMP13 positive area. **(E)** Heat map of biological nanoindentation of 28-day pretreated scaffolds. **(F)** Statistical results of effective Young’s modulus. **(G)** SP7 immunofluorescence staining image of the composite scaffold stimulated for 28 days *in vitro*. **(H)** Statistical results of percentage of SP7^+^ cells. Data were represented as the mean ± SD. *p < 0.05, **p < 0.01, ***p < 0.001, ****p < 0.0001.

After incubation for 28 days in a proliferative medium, immunofluorescence staining images for COL X (Figures 4A, B) and the corresponding statistical findings indicated that, compared to the CON group, the PSC group had significantly higher COL X expression as well as a wider distribution range. In contrast, PSC administration under chondrogenesis induction conditions considerably decreased COL X expression levels; however, their levels were significantly higher than those observed in the CON group. The trend of MMP13 was consistent with COL X, but there was no significant difference between the other groups except for a significant increase in CM compared to CON (Figures 4C, D). Interestingly, the hypertrophic chondrocyte like cells in the CM group also expressed COL X and MMP13 (Figures 4A, C).

In addition, scanning electron microscope imaging showed that hydrogel samples treated *in vitro* for 28 days produced many filamentous fibers, and the distribution of these fibers was more uniform under PSC (Supplementary Figure S2C). This phenotype might be connected to the regular cell arrangement that occurs as a result of increased cell polarity. The appearance and mechanical properties of the scaffold may vary as a result of cell-material interactions and ECM deposition. Therefore, we used a biological nanoindenter to examine its surface stiffness, with results indicating that the PSC administration alongside the proliferation medium greatly increased the Young’s modulus on average. The application of PSC under chondrogenesis induction conditions led to a reduction in the stiffness compared to CM treatment alone, which is consistent with the results for COL X expression and the appearance of the scaffolds (Figure 4E, F; Supplementary Figure S2D).

Our previous study reported that hypertrophic chondrocytes could transdifferentiate into osteoblasts during endochondral bone formation, revising the concepts of the chondrocyte-to-osteoblast lineage (Yang et al., 2014). SP7 (also known as Osterix) is an essential transcriptional activator for osteoblast differentiation, and it is one of the specific markers of osteoblasts. Immunofluorescence staining showed that about 20% of cells in CM and PSC + CM groups were SP7 positive, and there was no significant difference between the two groups. However, relatively few SP7-positive cells were observed under incubation with proliferation medium (Figures 4G, H). These results indicated that hypertrophic chondrocytes in the CM and CM + PSC groups had the potential to trans-differentiate into osteoblasts.

According to the above results, the ichondrocytes in the CM group undergo spontaneous hypertrophy as the treatment duration increased, while long-term (28 days) mechanical intervention inhibits hypertrophy and matrix deposition.

### 3.5 Ectopic outcome of composite scaffolds under biochemical/mechanical cue intervention

**FIGURE 5:**
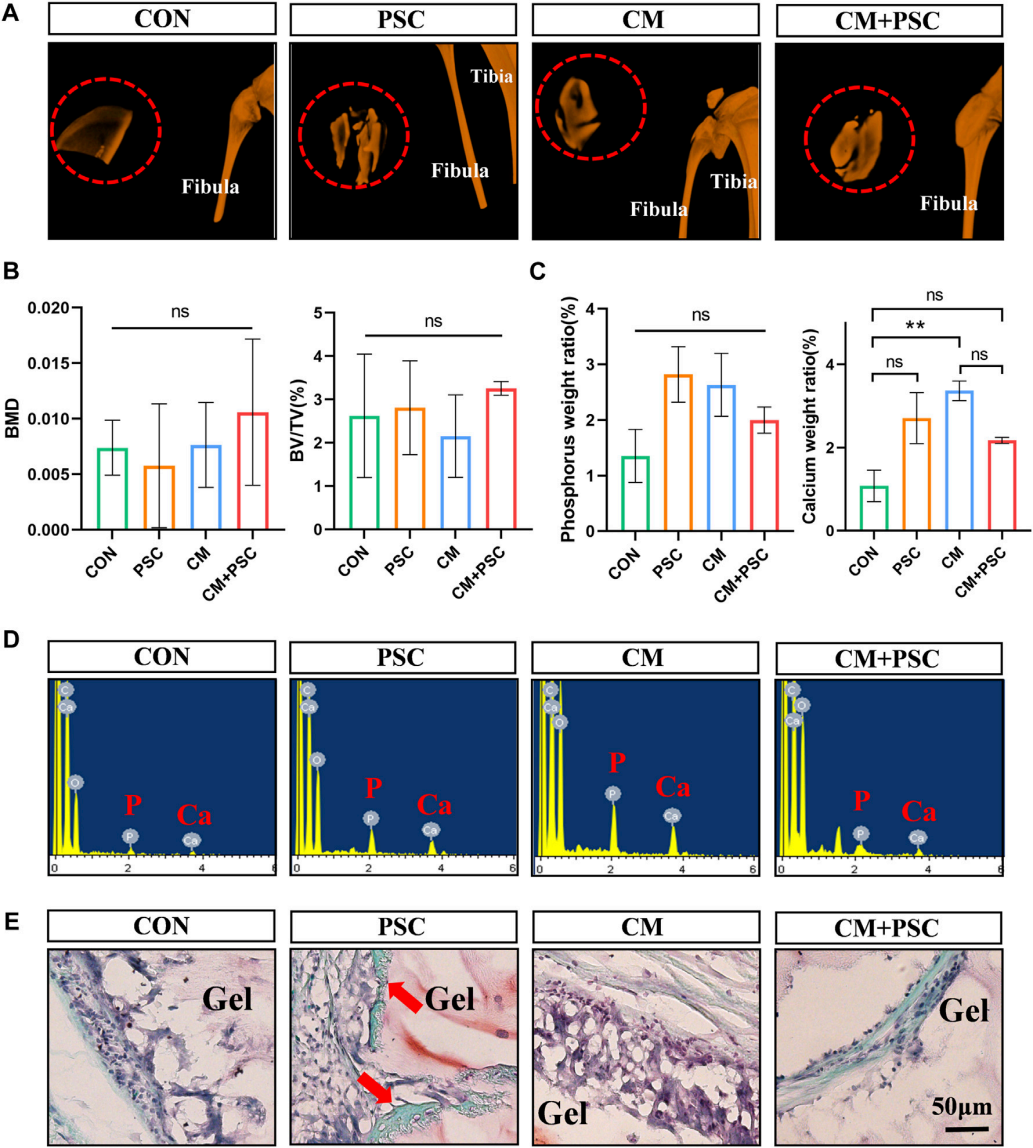
Ectopic outcome of composite scaffolds pretreated with biochemical/mechanical cues. **(A)** Micro-CT reconstruction image of composite scaffold in muscle pouch. The red dashed circle represents the hydrogel scaffold in the muscle pouches, and the image outside the red circle shows the tibia and fibula of the rat. **(B)** The statistical results of bone mineral density (BMD) and bone volume fraction (BV/TV). **(C,D)** EDX element analysis of composite scaffold at 10 weeks post operation. Statistical results and spectrograms of phosphorus and calcium contents. **(E)** Safranin O-fast green staining image of muscle pouch transplanted sample. The red arrow represents ectopic osteogenesis. Data were represented as the mean ± SD. **p < 0.01.

Single or synergistic biochemical/mechanical stimulation was applied to the composite scaffolds of the four groups (CON, PSC, CM, CM + PSC) mentioned above for 28 days. Subsequently, they were implanted into the rat femoral muscle pouches to further investigate whether the endochondral ossification process continued *in vivo*. The gross image revealed that the surface of the scaffold was covered by a coating of white substance that vanished entirely following decalcification, and is thus thought to be calcium salt deposition (Supplementary Figure S3A). We further conducted micro-CT analysis and reconstruction of the scaffolds in each group (Figures 5A, B), and found that calcium salt deposition was relatively uniform in both the PSC and CM + PSC groups. However, no significant differences were observed in terms of the bone mineral density (BMD) or the percentage of bone volume (BV/TV). In addition, after freeze-drying samples from each group, EDX elemental detection demonstrated that PSC treatment had no significant impact on the abundance of phosphorus and calcium ions, regardless of whether chondrogenic factors were used or not (Figures 5C, D). Furthermore, neither Safranin O-fast green nor HE staining showed any typical chondrocytes and signs of endochondral ossification. Interestingly, a small amount of new bone had formed in the PSC group (Figure 5E; Supplementary Figure S3B). It is well known that there are two forms of bone formation, intramembranous ossification and endochondral ossification. However, it was difficult to determine whether the new bone originates from the intramembranous or endochondral ossification lineage. These findings suggest that the pretreated scaffolds are unable to ectopically maintain the phenotype that was developed *in vitro*.

### 3.6 *In situ* outcome and bone repair effects of composite scaffolds under biochemical/mechanical cue intervention

**FIGURE 6:**
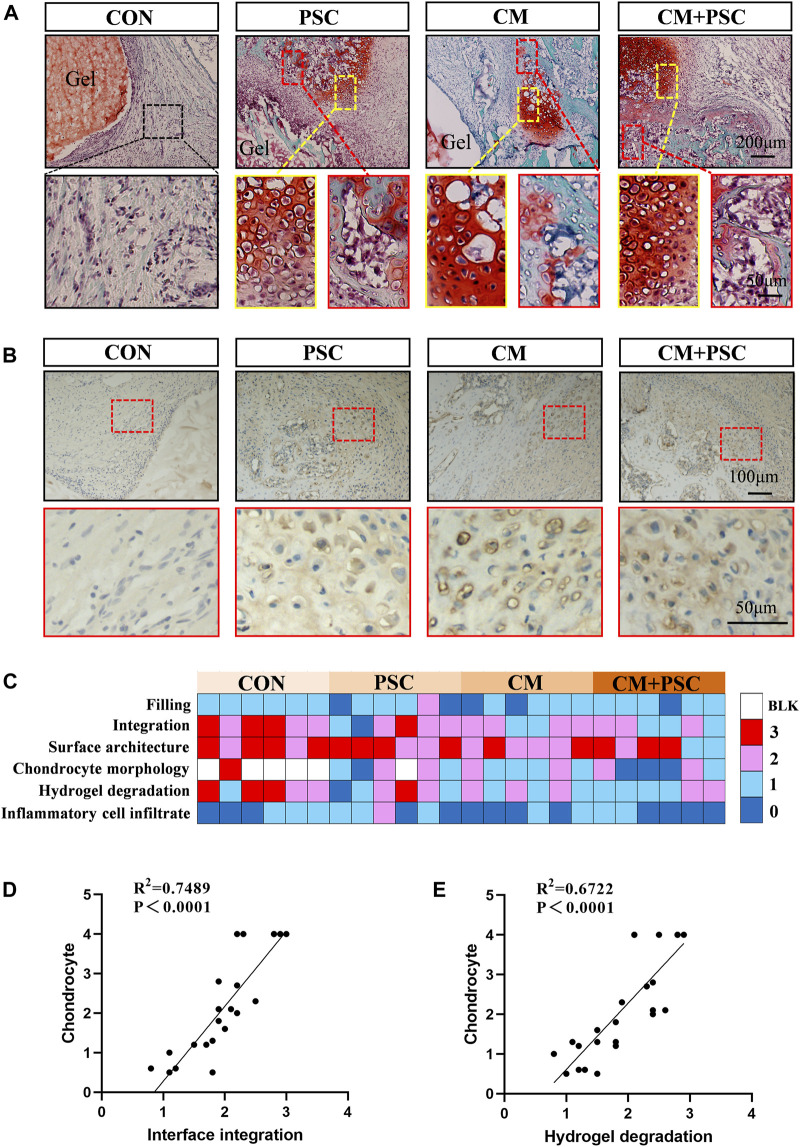
Chondrogenesis and endochondral bone formation of pretreated scaffolds after orthotopic transplantation. **(A)** Safranin O-fast green staining image of samples at 4 weeks post operation. The yellow box represents typical chondrocytes, and the red box represents endochondral bone formation. **(B)** Immunohistochemical staining image of COL Ⅱ. **(C)** Histological score of femoral condylar defect site at 4 weeks post operation. 0 represents a close to normal histological appearance and 3 represents a severe defect or completely unrepaired histological appearance. Blank box represents no chondrocyte distribution (BLK). **(D)** Pearson correlation analysis of chondrocyte and interface integration. **(E)** Pearson correlation analysis of chondrocyte and hydrogel degradation.

To observe whether the *in situ* microenvironment helps the pretreated scaffold retain its memory obtained *in vitro*, a rat femoral condyle defect model was constructed and scaffolds implanted into the defect site. The histological findings and healing status at the defect site were evaluated at 4 weeks and 10 weeks after surgery. Safranin O-fast green staining performed 4 weeks after surgery revealed that the interface between the material and the host bone in the CON group was filled with numerous fibrous tissues. However, a sizable amount of typical chondrocytes and endochondral osteogenic phenotypes were present at the interface, regardless of whether PSC and chondrogenic induction were performed separately or simultaneously (Figure 6A). COL Ⅱ immunohistochemical staining further confirmed the above phenotype (Figure 6B).

Results of the macroscopic histological appearance and semi-quantitative analysis of the defect repair indicated that the CM and CM + PSC groups outperformed the other two groups in terms of interfacial integration, chondrocyte morphology, and hydrogel degradation. Moreover, no significant differences were observed between the four groups in terms of inflammatory cell infiltration and defect filling (Figure 6C). On the other hand, the presence of chondrocytes was significantly positively linked with the development of the scaffold-host bone interface integration (Figure 6D) and the hydrogel degradation (Figure 6E).

**FIGURE 7:**
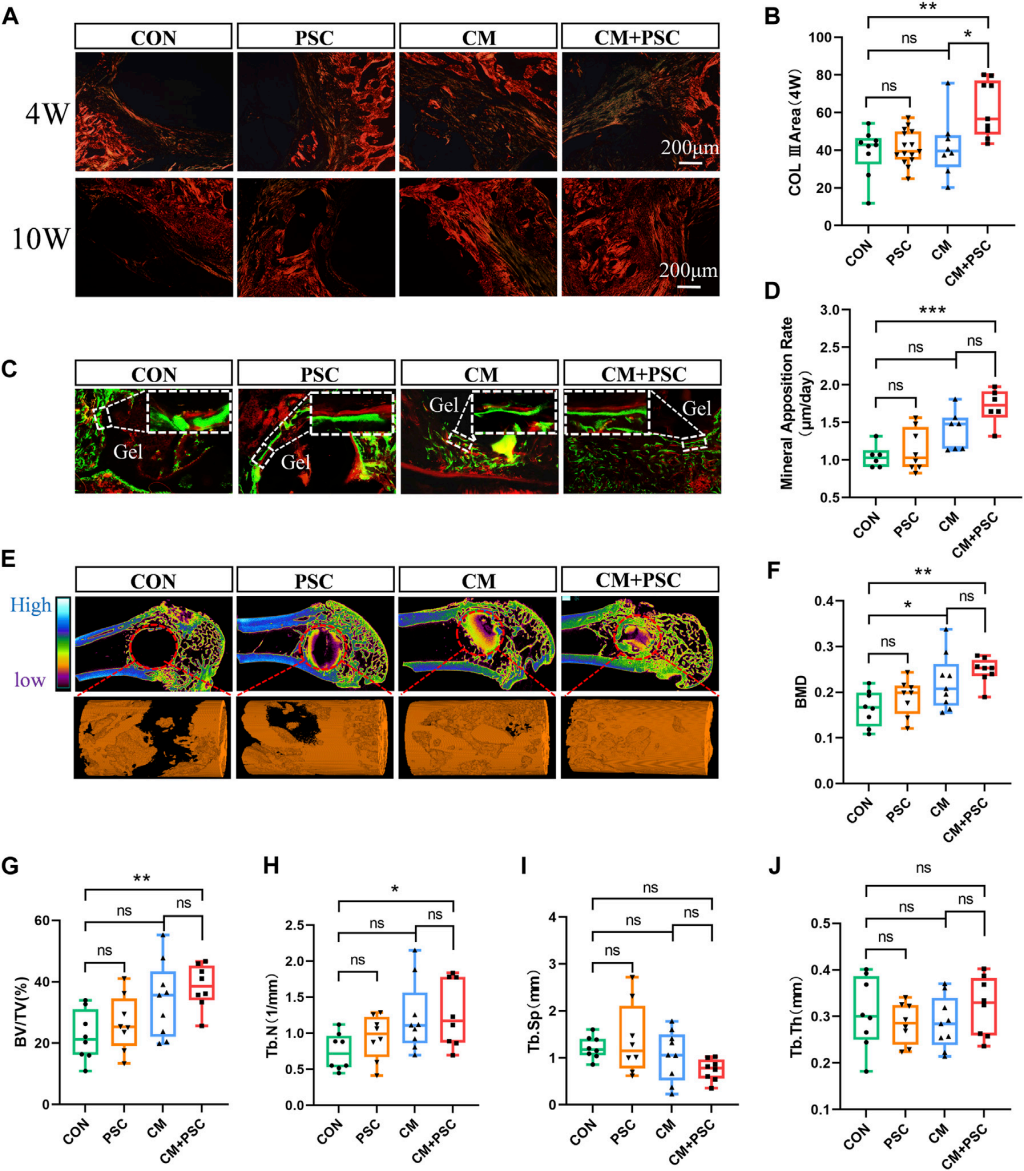
Role of composite scaffold pretreated with biochemical/mechanical cues on bone defect repair. **(A)** Sirius red staining image captured by polarized light microscope. The red and orange areas represent type I collagen, and the green elements represent type III collagen. **(B)** Statistical results of COL III area. **(C)** Fluorescence double labeling images of calcein and alizarin red. **(D)** Statistical analysis of mineral apposition rate. **(E)** Micro-CT reconstruction image of the bone defect site, the red dashed circle represents the ROI of the defect area, and the orange column is the 3D reconstruction image of new bone. **(F–J)** The statistical results of bone mineral density, bone volume fraction, bone trabecular number, bone trabecular separation and bone trabecular thickness. Data were represented as the mean ± SD. *p < 0.05, **p < 0.01, ***p < 0.001.

Defect repair is directly connected to the pace at which collagen filling occurs and the type of collagen present. Sirius red staining results showed that a significant amount of type Ⅲ collagen was distributed at the defect site in the CM + PSC group 4 weeks after surgery, but no significant difference in the type and distribution area of collagen was observed in the other three groups at this point. Type Ⅰ collagen comprised the majority 10 weeks after surgery in all groups (Figures 7A, B). Fluorescent double labeling with calcein and alizarin red was used to further assess the rate of new bone formation, with results revealing that using PSC with the proliferation medium did not particularly speed up the bone regeneration process. New bone formation was marginally accelerated by chondrogenesis induction, and the rate of mineral apposition was markedly accelerated by the combined effects of compressive loading and biochemical factors (Figures 7C, D).

Micro-CT was employed to perform three-dimensional reconstruction and quantitative analysis of the defect site in order to assess the overall impact of the scaffolds on bone repair in each pretreatment group. The findings demonstrated that the CM + PSC group had considerably greater BMD, BV/TV and Tb.N than the CON group. Nevertheless, no significant difference was observed in terms of Tb.Sp and Tb.Th. Although the application of PSC or CM alone has a certain positive effect on bone defect repair, the effect is limited (Figures 7E–J).

## 4 Discussion

This study investigated the effect of PSC on chondrogenic differentiation and bone defect repair by composite scaffolds loaded with BMSCs. The application of biochemical cues and mechanical loading *in vitro* was found to increase the chondrogenic differentiation potential of BMSCs in the short term (14 days), after which the ichondrocytes spontaneously underwent hypertrophy and maturation. Long-term (28 days) mechanical intervention was observed to prevent hypertrophy and mineralization of the ichondrocytes. However, ectopic implantation rendered the phenotype obtained *in vitro* unmaintainable, whereas considerable chondrocyte formation and evidence of endochondral ossification were observed 4 weeks after implantation into the bone defect site. Additional tests supported the conclusion that the synchronous biochemical/mechanical intervention of the composite scaffold significantly promoted the repair of bone defects.

Biochemical, mechanical, and genetic factors are all involved in determining the longitudinal growth of bone through endochondral bone formation (Killion et al., 2017). Mechanical loading is closely associated with the development and regeneration of bone-cartilage. Several studies have focused on the regulatory role and mechanism of dynamic loading on chondrogenic differentiation of stem cells (Fahy et al., 2017; Song, 2022; Ma et al., 2022). Mechanical cues control chondrocyte homeostasis or hypertrophy and mineralization, depending on the timing of the load application (McDermott et al., 2021). Recently, Professor Jiang Q. and others discovered that beginning dynamic compression early in the chondrogenesis induction period (day 1) decreased the expression of some chondrocyte markers, while mechanical loading at a later time (day 21) could maintain the phenotypes of the chondrocytes (Ge et al., 2021). The specific biological impacts of mechanical intervention may also depend on loading pattern factors such as the degree of strain. At present, mechanical research that uses biological scaffolds as stem cell carriers mostly involves strain over the range 5%–20% (Aisenbrey and Bryant, 2016; Kowsari-Esfahan et al., 2018; Cao et al., 2019; Wang et al., 2019). For example, a study by R Kowsari-Esfahan discovered that 10% compressive strain can be considered an optimal stimulation factor for the chondrogenic differentiation of adipose derived stem cells (Kowsari-Esfahan et al., 2018). However, under physiological conditions, bone and cartilage are highly mechanically sensitive, and their microstructure may be disrupted by extensive compressive strain. Unfortunately, static compression patterns characterized by micro-strain are rarely reported.

We designed a simple static compression pattern, with the scaffold used producing a strain of only 0.5% at most when stressed. The periodic loading and unloading ensures that the cells in the scaffold obtain sufficient nutrients and oxygen. The survival and proliferation state of the cells in the scaffold was validated by the results of HE and Ki67 staining 7 days after loading. However, whether the BMSCs are capable of sensing micro-strain mechanics remains unknown, although a study suggests that cells may be able to react to and transduce micro-mechanics, according to a recent report on a piconewton-range mechanosensitive receptor (Sloas et al., 2023). BMSCs were chosen as the research subject in this study primarily because of their potent chondrogenic potential and effective mechanical signal perception (Wang et al., 2022a; Sun et al., 2022). The results of the cell polarity and chondrogenic differentiation indicate that the BMSCs did react to micro-strain mechanical stimulation. Previous studies suggested that stress-induced polar distribution of the cytoskeleton regulates cell migration and adhesion (Lim Lam et al., 2021; Uray and Uray, 2021). The relatively uniform collagen fibers and ectopic calcium salt deposition within the scaffold may be explained by the fact that PSC leads to polarization and migration of BMSC, and then uniformly distributed in the hydrogel.

A few cells started to express SOX9 after 14 days of PSC treatment *in vitro*, demonstrating that PSC had a specific inducing effect on the chondrogenic differentiation of BMSCs. These results are consistent with the viewpoints summarized in Fahy N’s review (Fahy et al., 2017). However, chondrogenic induction alone or in combination with PSC for 14 days dramatically raised the proportion of SOX9-positive cells, indicating that biochemical cues are important for the chondrogenic differentiation of BMSCs. At 28 days after chondrogenic induction, suspected hypertrophic chondrocytes were discovered in the samples, showing a propensity for spontaneous hypertrophy and maturation of the ichondrocytes. However, the positive expression area of COL X and average Young’s modulus in the CM + PSC group were significantly lower than those in the CM group, indicating that prolonged static compression might prevent chondrocyte hypertrophy and aid in preserving the phenotype and homeostasis. The above results indicate that biochemical clues and PSC may play regulatory roles at different stages of chondrocyte differentiation, respectively. Biochemical cues are the main regulators during the differentiation of BMSCs into chondrocytes, and PSC plays an important role in inhibiting the process of ichondrocyte hypertrophy and maturation.

According to a study by Price, cells have the potential to retain the mechanical history in the microenvironment to produce long-term mechanical memory (Price et al., 2021). But it is not clear whether the “biochemical-mechanical memory” acquired *in vitro* can be inherited and developed in the *in vivo* microenvironment. In our study, no ectopic chondrogenesis was observed after implantation of the pretreated scaffolds into the rat femoral muscle pouches. Muscle is recognized as the second secretory organ in the human body (Tagliafico et al., 2022); therefore, its peculiar microenvironment may be able to prevent or reverse the established fate of exogenous cells, which is one possible explanation for this phenomenon. Correspondingly, a study in which pre-induced osteogenic biomaterial with apertures at both ends was implanted into the back subcutaneous area of mice in 2014 by Donald E. Ingber et al. showed that a significant amount of fat cells filled the inside of the material (Torisawa et al., 2014), implying that the ectopic microenvironment had a suppressive effect on osteogenesis. Recently, Prof. Cao Y.L. and Zhou G.D. published a paper detailing the impact of the authentic ear cartilage and articular cartilage niche on the differentiation fate of MSCs and the type of regenerated cartilage, with results indicating that the primary determinant for the type of cartilage regenerated was the local cartilage niche (Hou et al., 2022). These studies highlight the *in situ* induction and assimilation effects of local niches.

The outcomes of pretreated scaffolds in the *in-situ* microenvironment of bone defects were also investigated in this study. Four weeks after operating, typical chondrocytes and endochondral new bone were visible in samples that had been subjected to biochemical induction or mechanical stimulation, either alone or in combination. The fact that no chondrocytes were observed in the control group suggests that the strong or weak chondrogenic memory endowed *in vitro* is preserved and developed in the bone defect niche. Several advantageous outcomes were observed as a result of the production of chondrocytes, including improvement of the host bone-material interface integration and faster scaffold degradation. In addition, we found much higher type III collagen content at the defect site of the CM + PSC group than that of the other three groups, suggesting that pretreatment with CM + PSC may aid in the creation and deposition of regenerative substances at the defect site, because type III collagen has a positive regulatory role in bone repair (Cheng et al., 2018). The further evaluation results for bone regeneration likewise supported the benefit of CM + PSC pretreatment for bone repair, showing that biochemical cues and mechanical loading work together to promote endochondral bone formation, much like the process of bone development.

In summary, a PSC model characterized by micro-strain was designed to uncover the regulatory effects of biochemical factors (TGF-β3 and ITS, etc.,) and mechanical loading on BMSC differentiation. We further described the adaptation process of “biochemical-mechanical memory” to the *in vivo* microenvironment. Although the underlying mechanism of the micro-strain mechanical loading regulating chondrogenic differentiation of BMSCs remains to be elucidated, our results provide a candidate strategy for the preparation and application of developmental scaffolds based on endochondral bone formation.

## Data Availability

The original contributions presented in the study are included in the article/Supplementary Material, further inquiries can be directed to the corresponding authors.
