# Supplementary material for: Periodic static compression of micro-strain pattern regulates endochondral bone formation
Source: Front Bioeng Biotechnol. 2024 Mar 27;12:1356135. doi: 10.3389/fbioe.2024.1356135 (PMC11004279; doi:10.3389/fbioe.2024.1356135)
Supplement: Supplementary file 1 [file DataSheet1.DOCX]

**Supporting Information**

Periodic static compression of micro-strain pattern regulates endochondral bone formation

Pengzhen Cheng^1,2,3,4†^, Xueyi Zhao^3†^, Meige Han^1,2†^, Yaping Zhuang^6†^, Fenru Ning^7^, Yaqian Hu^3^, Weiguang Lu^3^, Sheng Miao^3^, Chengxiang Zhao^3^, Liyuan Jia^1^, Xue Hao^2,4^, Meng Sun^1^, Junxiang Wang^1,2^, Fulin Chen^1,4,5*^, Liu Yang^3*^and Qiang Jie^2,4,5*^


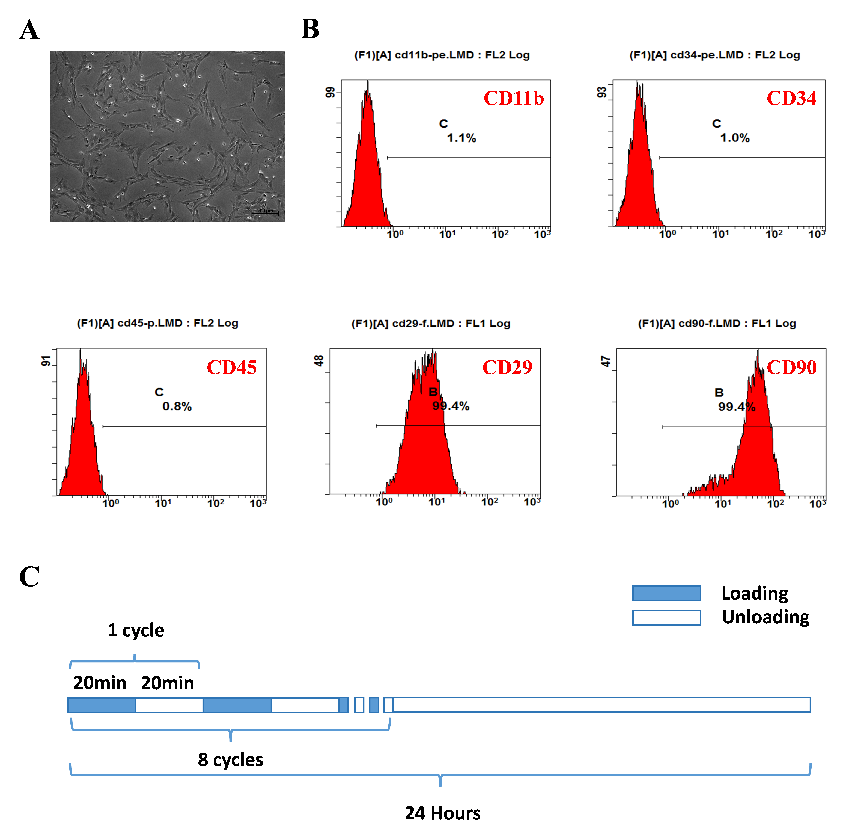


**FIGURE S1** Morphology and identification results of rat BMSCs. (**A**) Cell morphology image. (**B**) Identification of specific markers of BMSCs by flow cytometry (CD11b-, CD34-, CD45-, CD29+, CD90+). (**C**) Procedures for the mechanical loading experiments. A straightforward in vitro loading test model, which continually applied a static compression load to a scaffold in a 24-hole plate using a titanium alloy block, was created, with one cycle comprising 20 min loading and 20 min unloading. A total of 8 cycles were performed each day.


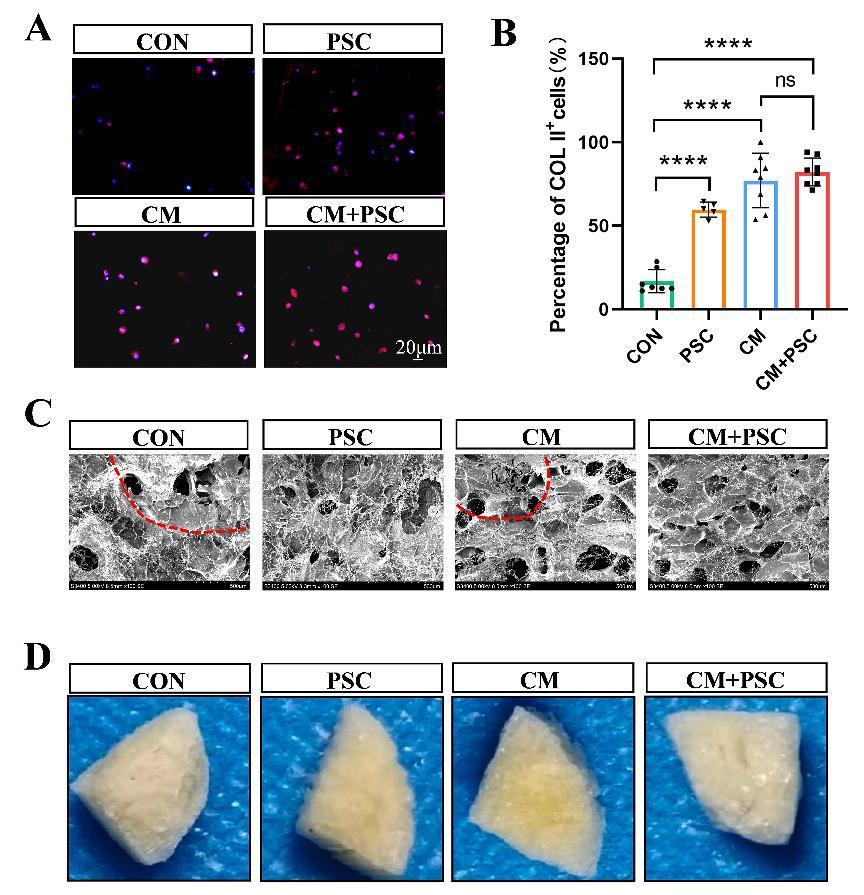


**FIGURE S2** Effects of biochemical/mechanical cues on matrix deposition of BMSCs-loaded scaffolds. (**A**) COLⅡ immunofluorescence staining image of the composite scaffold stimulated for 14 days *in vitro*. (**B**) Statistical results of percentage of COLⅡ^+^ cells. (**C**) SEM images of composite scaffolds pretreated for 28 days,the inner side of the red arc is the region without extracellular matrix deposition. (**D**) Appearance of freeze-dried scaffold after biochemical/mechanical pretreatment for 28 days.Data were represented as the mean ± SD, ****p < 0.0001.


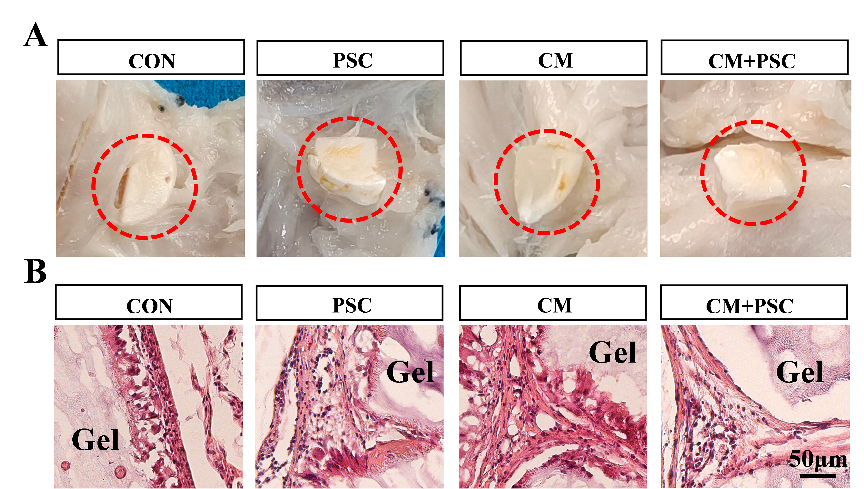


**FIGURE S3** Ectopic outcome of composite scaffolds pretreated with biochemical/mechanical cues. (**A**) General appearance of composite scaffolds in muscle pouch at 10W post operation. (**B**) HE staining image of muscle pouch transplanted sample.
